# Nonlinear Association Between NHHR and Suicide Attempts in First‐Episode Untreated Depression With a Threshold Effect

**DOI:** 10.1155/da/4634746

**Published:** 2026-05-11

**Authors:** Xiaofang Shang, Diwen Shen, Ya-nan Sun, Xiao-E Lang, Junjun Liu, Xiangyang Zhang

**Affiliations:** ^1^ Department of Psychiatry, Nanjing Medical University Affiliated Brain Hospital, Nanjing, 210029, China, c-nbh.com; ^2^ Department of Clinical Psychology, Nanjing Drum Tower Hospital, Affiliated Hospital of Medical School, Nanjing University, Nanjing, 210008, China, nju.edu.cn; ^3^ Department of Clinical Laboratory, Nanjing Brain Hospital, Nanjing, 210029, China, njmu.edu.cn; ^4^ Department of Psychiatry, First Hospital of Shanxi Medical University, Taiyuan, 030000, China, sxmu.edu.cn; ^5^ Department of Psychiatry, Nanjing Meishan Hospital, Nanjing, 210041, China; ^6^ Hefei Fourth People’s Hospital, Anhui Mental Health Center, Affiliated Psychological Hospital of Anhui Medical University, Hefei, 230022, Anhui, China, ahmhcentre.com

**Keywords:** major depressive disorder, NHHR, nonlinear association, suicide attempts, threshold effect

## Abstract

**Objective:**

To investigate, with an emphasis on possible nonlinearity, the relationship between suicide attempts (SAs) and the ratio of non‐high‐density lipoprotein cholesterol to high‐density lipoprotein cholesterol ratio (NHHR) in patients with first‐episode untreated (FEU) major depressive disorder (MDD).

**Methods:**

A total of 1718 FEU MDD were included in this cross‐sectional study. NHHR was calculated from the fasting lipid profiles. Multivariable logistic regression was used to assess the independent association between the NHHR and SA across the three adjusted models. Restricted cubic splines (RCSs) and two‐piecewise logistic regression were applied to characterize the dose–response relationship and identify the inflection point.

**Results:**

SA occurred in 346 (20.1%) patients. SA prevalence increased progressively across the NHHR tertiles: 10.82% (T1), 17.13% (T2), and 32.46% (T3) (*p* < 0.001). Higher SA risk was independently linked to each unit increase in NHHR in the fully adjusted model (OR = 1.08, 95% CI: 1.01–1.16). At NHHR = 5.76, a significant nonlinear threshold effect was found; below the limit, every unit increase in NHHR was associated with a 27% greater likelihood of SA (OR = 1.27, 95% CI: 1.12–1.43, *p* < 0.001), while outside the limits, no significant association emerged (OR = 0.85, *p* = 0.092).

**Conclusions:**

NHHR was independently and nonlinearly associated with SA in patients with FEU MDD, with a clinically meaningful threshold of 5.76. As a readily available lipid‐derived marker calculable from standard fasting panels, NHHR may serve as a practical tool for early suicide risk stratification in clinical settings.

## 1. Background

Major depressive disorder (MDD), one of the most widespread and crippling mental illnesses, is characterized by a constellation of physical and cognitive symptoms, anhedonia, and persistently low mood [1]. According to the World Health Organization, MDD is a major cause of disability worldwide and has a significant impact on those who are affected, their families, and healthcare systems [2]. Suicide attempt (SA), characterized by self‐harming behavior coupled with at least some intent to die, is a serious and potentially fatal side effect of MDD. Accumulating evidence indicates that individuals with MDD face a markedly elevated risk of SA, with established risk factors including a family history of suicide, negative life events, and suicidal ideation [3]. Furthermore, patients with MDD with suicidal ideation or behavior exhibit greater psychological distress, more severe social functional impairment, and poorer quality of life than those without [4]. Therefore, the early identification of individuals at elevated SA risk constitutes a matter of paramount clinical urgency. Within this population, first‐episode untreated (FEU) patients occupy a uniquely informative position: having not yet received pharmacological intervention, their biological profiles reflect the intrinsic pathophysiology of the disorder, which is unconfounded by antidepressant medications [5]. This treatment‐naïve state renders them an optimal cohort for investigating the objective biological markers of SA risk. Nevertheless, despite the pressing clinical need, reliable and readily accessible biomarkers for SA risk stratification in this population remain scarce. Emerging evidence suggests that metabolic dysregulation, encompassing lipid metabolism abnormalities and atherosclerosis‐related pathways, may be linked to the pathophysiology of both MDD and associated suicidal behavior [6], providing a rationale for exploring novel lipid‐based indices as potential risk stratification tools.

Considerable evidence has demonstrated that patients with MDD exhibit widespread disturbances in their lipid metabolism. A systematic review of drug‐naïve patients with MDD reported significantly lower total cholesterol levels compared with healthy controls, particularly in male‐predominant samples [7]. Beyond single lipid markers, lipidomic profiling has revealed that MDD is associated with alterations in up to 97 distinct lipid species, implicating pathways related to inflammation, lipid peroxidation, and mitochondrial dysfunction [8]. Conventional lipid indices, including TC, LDL‐c, and HDL‐c, assessed in isolation, capture only a single dimension of lipid metabolism and may fail to adequately reflect the dynamic equilibrium between atherogenic and antiatherogenic lipoprotein fractions. To address this limitation, the non‐high‐density lipoprotein cholesterol to high‐density lipoprotein cholesterol ratio (NHHR) has been proposed as a new composite lipid metric that simultaneously incorporates information from both antiatherogenic (HDL‐c) and atherogenic (non‐HDL‐c) components [9]. This integrative approach provides a more comprehensive assessment of atherogenic lipid burden than any single fraction alone. The NHHR has already demonstrated robust associations with cardiovascular disease risk [10], hypertension [11], and metabolic disorders across diverse populations. Notably, its clinical relevance has been extended to psychiatric domains; elevated NHHR has a favorable correlation with depression symptoms in the general population [9] and with suicidal ideation following full multivariable adjustment [12]. NHHR has been identified as an independent predictor of poststroke depression [13] and sex‐specific alterations in HDL‐related indicators, including NHHR, have been documented in patients with MDD and bipolar disorder [14]. Importantly, NHHR is derived directly from standard fasting lipid panels without the need for additional testing, thus conferring exceptional clinical accessibility and scalability. To date, no study has specifically examined the correlation between SA and NHHR in patients with FEU MDD, nor has any investigation explored whether this relationship follows a nonlinear trajectory.

The absence of such evidence reflects the broader methodological and population‐level limitations in the existing literature. The few studies examining NHHR in psychiatric contexts have been conducted in heterogeneous general population samples, with substantial confounding attributable to antidepressant use, comorbid conditions, and lifestyle factors [15]. Critically, no prior study has focused exclusively on FEU patients with MDD, a population in which biological signals remain uncontaminated by pharmacological treatment, permitting the investigation of the disorder’s natural course in its most unconfounded form. Moreover, objective and readily accessible biomarkers for suicide risk stratification remain scarce in this vulnerable group, representing a critical gap in clinical practice [16]. Previous investigations of lipid biomarkers and suicidal behavior in MDD have predominantly employed conventional linear analytical frameworks, which presuppose a uniform dose–response relationship across the full exposure range [17], which may obscure clinically meaningful threshold effects or inflection points at which the risk relationship fundamentally changes in character. To address these gaps, this cross‐sectional study enrolled FEU patients with MDD and aimed to investigate the independent association between SA and NHHR, with particular attention to potential nonlinear relationships and threshold effects.

## 2. Methods

### 2.1. Subjects

This cross‐sectional study was conducted between September 2016 and December 2018 at the First Hospital of Shanxi Medical University, China. The mean age of the 1718 consecutive FEU patients with MDD who were enrolled was 34.87 ± 12.43 years (range: 18–60 years), with 1130 females (65.8%) and 588 males (34.2%) making up the total. The MDD diagnoses were independently established by two qualified psychiatrists.

The inclusion criteria were as follows: (1) meeting the DSM‐IV‐TR diagnostic criteria for MDD [18]; (2) not having previously been exposed to antidepressants; (3) presenting with an acute first depressive episode; (4) aged 18–60 years and being Chinese Han; and (5) having a total score of ≥24 on the 17‐item Hamilton Depression Rating Scale (HAMD‐17).

The exclusion criteria were as follows: (1) alcohol or substance use disorder (tobacco use excluded); (2) current pregnancy or lactation; (3) serious medical comorbidities, including malignancy, active or ongoing infection, stroke, traumatic brain injury, or epilepsy; (4) concurrent DSM‐IV‐TR Axis I diagnosis, including schizophrenia, schizoaffective disorder, or bipolar disorder; (5) inability to complete the clinical interview due to acute clinical deterioration; or (6) refusal to provide informed consent.

With ethical approval from the IRB of the First Hospital of Shanxi Medical University (Number 2016‐Y27), this study was conducted in accordance with the Declaration of Helsinki, and written informed consent was obtained from all participants prior to enrollment.

### 2.2. Sociodemographic and Anthropometric Data

Using a purpose‐designed questionnaire administered through structured face‐to‐face interviews, the following variables were recorded: age (years), sex, educational attainment, marital status, illness duration (months), and age at symptom onset (years). Body mass index (BMI), SBP, and DBP were also assessed.

The fasting lipid profile, including TC and high‐density lipoprotein cholesterol (HDL‐c) levels, was assessed following a standardized minimum fasting duration of at least 8 h. The NHHR was subsequently calculated as follows: NHHR = (TC − HDL‐c)/HDL‐c [9].

### 2.3. Clinical Assessment

The WHO/EURO Multicenter Study on Parasuicide’s “Have you ever attempted suicide during the current depressive episode?” question was used in structured face‐to‐face interviews to determine SA [19], which was defined as self‐harming behavior coupled with at least some intent to end one’s life. Additional details were obtained regarding the number, method, and date(s) of each attempt. Among the 346 patients with a history of SA, 317 reported one attempt, 26 reported two attempts, two reported three attempts, and one reported four attempts. The methods employed included jumping from heights, self‐cutting, traffic‐related acts, hanging, gas inhalation, charcoal burning, and drowning.

A Chinese translation of the HAMD‐17, which yields a total score ranging from 0 to 52, was used to assess the severity of depressive symptoms. Higher scores indicated more severe symptoms. The 14‐item Hamilton Anxiety Rating Scale (HAMA) in Chinese was used to measure the intensity of anxiety symptoms. The results showed a total score ranging from 0 to 56. Both tools have been extensively used in clinical research and have proven valid and reliable in earlier investigations [20, 21]. All psychiatrists underwent standardized training for HAMD‐17 and HAMA administration prior to data collection. Interrater reliability was confirmed by ICCs exceeding 0.80 for both scales, and all raters were blinded to the participants’ diagnoses and study hypotheses throughout.

### 2.4. Statistical Analysis

Normality was assessed using the Shapiro–Wilk test. Continuous variables are presented as mean ± standard deviation (SD), and categorical variables as frequencies and proportions. Baseline characteristics were stratified by NHHR tertile (T1–T3) and compared using one‐way ANOVA or the chi‐square test. Univariate logistic regression was performed to screen for clinical and sociodemographic variables for their individual associations with SA. Variables that achieved statistical significance in the univariate analyses were subsequently entered into the multivariable logistic regression models. Three models assessed the NHHR–SA association: Model 1, unadjusted; Model 2, adjusted for age and sex; and Model 3, fully adjusted for age, education, illness duration, HAMD‐17, HAMA, SBP, and DBP. NHHR was analyzed as both a continuous and categorical variable (tertiles; T1 as reference). The results are reported as ORs with 95% CIs. Restricted cubic spline (RCS) regression (four knots: 5th, 35th, 65th, and 95th percentiles) was used to assess the nonlinearity. A two‐piecewise logistic regression model was then applied to identify the inflection point determined by minimizing model deviance. Model superiority over single linear regression was confirmed using the log‐likelihood ratio test.

Analyses were performed in SPSS (v25.0) and R (v4.3.0) using the rms and segmented packages. All tests were two‐tailed; *p* < 0.05 was considered significant.

## 3. Results

### 3.1. Baseline Characteristics Across NHHR Tertiles

A total of 1718 FEU patients with depression were enrolled in this cross‐sectional study. SA, defined as self‐injurious behavior with at least some intent to die, was identified in 346 patients, yielding a prevalence of 20.1%. NHHR, as a composite lipid metabolism indicator, had a mean value of 3.66 ± 1.93, ranging from 0.36 to 13.66. Notably, the proportion of patients with SA increased progressively across the NHHR tertiles from 10.82% in the low tertile to 17.13% in the middle tertile and 32.46% in the high tertile (Table 1). The sex distribution and educational level of the three tertile groups were comparable (both *p* > 0.05). In contrast, TC, HDL‐c, SBP, DBP, HAMD, HAMA, duration of illness, age, marital status, and SA differed significantly across the NHHR tertiles (all *p* < 0.001), and BMI also showed a statistically significant difference (*p* = 0.034).

**Table 1:** Baseline characteristics of patients stratified by NHHR tertiles.

Variables	NHHR tertile			
	Low	Middle	High	*p*‐Value
*N*	573	572	573	
Age (years)	33.15 ± 12.36	35.21 ± 11.95	36.25 ± 12.78	<0.001
Sex				0.532
Male	199 (34.73%)	203 (35.49%)	186 (32.46%)	
Female	374 (65.27%)	369 (64.51%)	387 (67.54%)	
Marital status				<0.001
Single	202 (35.25%)	146 (25.52%)	154 (26.88%)	
Married	371 (64.75%)	426 (74.48%)	419 (73.12%)	
Education				0.168
Junior high school	118 (20.59%)	136 (23.78%)	159 (27.75%)	
Senior high school	261 (45.55%)	257 (44.93%)	242 (42.23%)	
College	157 (27.40%)	151 (26.40%)	141 (24.61%)	
Postgraduate	37 (6.46%)	28 (4.90%)	31 (5.41%)	
Duration of illness (months)	5.49 ± 4.14	6.56 ± 5.13	6.88 ± 4.77	<0.001
HAMD	28.87 ± 2.81	30.27 ± 2.70	31.75 ± 2.59	<0.001
HAMA	19.92 ± 3.21	20.65 ± 3.34	21.82 ± 3.59	<0.001
BMI (kg/m^2^)	24.26 ± 1.87	24.30 ± 1.93	24.54 ± 1.96	0.034
SBP (mmHg)	115.39 ± 10.94	119.02 ± 10.36	124.04 ± 9.64	<0.001
DBP (mmHg)	74.32 ± 6.59	75.76 ± 6.61	77.77 ± 6.59	<0.001
TC (mmol/L)	4.36 ± 0.77	5.18 ± 0.72	6.21 ± 0.91	<0.001
HDL‐c (mmol/L)	1.45 ± 0.22	1.27 ± 0.18	0.95 ± 0.20	<0.001
SA				<0.001
No	511 (89.18%)	474 (82.87%)	387 (67.54%)	
Yes	62 (10.82%)	98 (17.13%)	186 (32.46%)	

Abbreviations: BMI, body mass index; DBP, diastolic blood pressure; HAMA, Hamilton Anxiety Rating Scale; HAMD, Hamilton Depression Rating Scale; HDL‐c, high‐density lipoprotein cholesterol; NHHR, non‐high‐density lipoprotein cholesterol to high‐density lipoprotein cholesterol ratio; SA, suicide attempt; SBP, systolic blood pressure; TC, total cholesterol.

### 3.2. Univariate Logistic Regression Analysis for SA

Univariate logistic regression (Table 2) revealed that age, illness duration, HAMD, HAMA, SBP, DBP, and NHHR were positively associated with SA, whereas senior high school and college education (vs. junior high) were inversely associated (all *p* < 0.05). Sex, marital status, postgraduate education, and BMI were not significant (all *p* > 0.05).

**Table 2:** Univariate logistic regression analysis for SA.

Variables	Mean ± SD, *N* (%)	OR (95% CI)	*p*
Age (years)	34.87 ± 12.43	1.01 (1.00, 1.02)	0.036
Sex			
Male	588 (34.23%)	1.0	
Female	1130 (65.77%)	1.11 (0.86, 1.43)	0.416
Marital status			
Single	502 (29.22%)	1.0	
Married	1216 (70.78%)	1.11 (0.86, 1.45)	0.420
Education			
Junior high school	413 (24.04%)	1.0	
Senior high school	760 (44.24%)	0.71 (0.53, 0.95)	0.022
College	449 (26.14%)	0.69 (0.50, 0.96)	0.027
Postgraduate	96 (5.59%)	1.04 (0.62, 1.74)	0.872
Duration of illness (months)	6.31 ± 4.73	1.03 (1.01, 1.06)	0.005
HAMD	30.30 ± 2.94	1.36 (1.30, 1.43)	<0.001
HAMA	20.80 ± 3.47	1.36 (1.31, 1.42)	<0.001
BMI (kg/m^2^)	24.37 ± 1.92	0.98 (0.93, 1.05)	0.622
SBP (mmHg)	119.48 ± 10.91	1.06 (1.04, 1.07)	<0.001
DBP (mmHg)	75.95 ± 6.74	1.07 (1.06, 1.09)	<0.001
NHHR	3.66 ± 1.93	1.29 (1.22, 1.37)	<0.001

Abbreviations: BMI, body mass index; DBP, diastolic blood pressure; HAMA, Hamilton Anxiety Rating Scale; HAMD, Hamilton Depression Rating Scale; NHHR, Non‐high‐density lipoprotein cholesterol to high‐density lipoprotein cholesterol ratio; SA, suicide attempt; SBP, systolic blood pressure.

### 3.3. NHHR as Continuous Variable and Tertiles and SA Across Multiple Logistic Regression Models

Table 3 shows that NHHR was significantly associated with SA as a continuous variable across all three models (Model 1: OR = 1.29; Model 2: OR = 1.29; Model 3: OR = 1.08; all *p* < 0.05). For tertile analysis, T3 (vs. T1) was significant in all models (Model 1: OR = 3.96; Model 2: OR = 3.89; Model 3: OR = 1.85; all *p* < 0.05). T2 was significant in Models 1–2 but not Model 3 (OR = 1.20, *p* = 0.337).

**Table 3:** Logistic regression for SA in different models.

Variables	Model 1		Model 2		Model 3	
	OR (95% CI)	*p*‐Value	OR (95% CI)	*p*‐Value	OR (95% CI)	*p*‐Value
NHHR	1.29 (1.22, 1.37)	<0.001	1.29 (1.22, 1.37)	<0.001	1.08 (1.01, 1.16)	0.035
NHHR tertile						
Low T1	1.0		1.0		1.0	
Middle T2	1.70 (1.21, 2.40)	0.002	1.68 (1.20, 2.37)	0.003	1.20 (0.82, 1.76)	0.337
High T3	3.96 (2.89, 5.44)	<0.001	3.89 (2.83, 5.34)	<0.001	1.85 (1.28, 2.68)	0.001

*Note:* Nonadjusted model, adjust for: none. Adjust I model adjust for: age; gender. Adjusted II model adjusted for age, education, illness duration, HAMD‐17, HAMA, SBP, and DBP.

### 3.4. Nonlinear Relationship Between NHHR and SA

**Figure 1:**
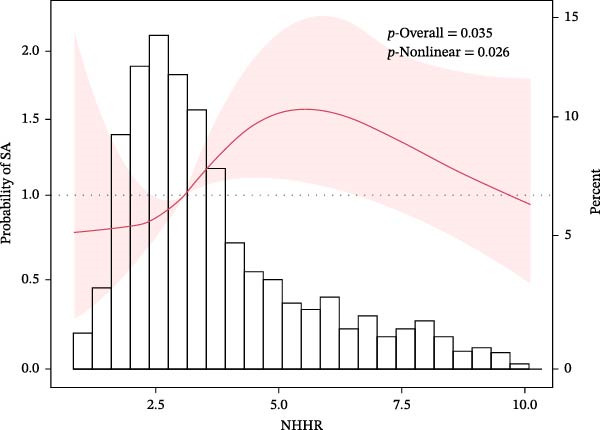
The relationship between NHHR and the probability of suicide attempts. A nonlinear relationship between NHHR and the probability of suicide attempts was observed after adjusting for age, education, illness duration, HAMD‐17, HAMA, SBP, and DBP (log‐likelihood ratio test, *p* = 0.026).

Curve fitting revealed a nonlinear NHHR–SA association (Figure 1). Two‐piecewise logistic regression (Table 4) identified an inflection point at NHHR = 5.76. Below 5.76, NHHR was positively associated with SA (OR = 1.27, *p* < 0.001); however, at or above 5.76, the association was not significant (OR = 0.85, *p* = 0.092). The two‐piecewise model outperformed the single‐segment model (log‐likelihood ratio test, *p* = 0.026).

**Table 4:** The results of two‐piecewise logistic regression model.

Inflection points of NHHR	Effect size (OR)	95% CI	*p*
Inflection point (*K*)	5.76		
<5.76	1.27	1.12, 1.43	<0.001
≥5.76	0.85	0.71, 1.03	0.092
Log‐likelihood ratio test			0.026

*Note:* Effect: suicide attempts; cause: NHHR; adjusted for age, education, illness duration, HAMD‐17, HAMA, SBP, and DBP.

## 4. Discussion

In this cross‐sectional study of 1718 FEU patients with MDD, the NHHR was independently and positively associated with SA after full adjustment for potential confounders. Tertile analysis demonstrated a dose‐dependent gradient, with SA proportions rising from 10.82% (T1) to 17.13% (T2) and 32.46% (T3); T3 conferred 85% greater odds of SA than T1. RCS and two‐piecewise regression identified a nonlinear NHHR–SA relationship, with an inflection point at 5.76, below which each unit increase in NHHR was associated with 27% higher odds of SA. To our knowledge, this is the first study to examine NHHR and SA in FEU MDD, extending prior evidence linking NHHR to depressive symptoms and suicidal ideation to the more severe outcomes of SA.

The observed association between NHHR and SA in the present study may be attributable to several interrelated biological pathways through which atherogenic lipid dysregulation exerts detrimental effects on the brain. First, from an inflammatory standpoint, elevated NHHR reflects a shift in the lipid milieu toward an atherogenic profile, wherein rising non‐HDL‐c promotes vascular inflammation as well as endothelial dysfunction, while concurrently reducing HDL‐c compromises the anti‐inflammatory and antioxidant properties normally conferred by this lipoprotein fraction [22]. Neuroinflammation, mediated by microglial activation, upregulation of proinflammatory cytokines, and disruption of neurovascular barrier integrity, is now recognized as a fundamental pathophysiological driver of MDD, implicating neuroimmune, metabolic, and oxidative‐nitrosative stress pathways underlying the development and severity of depressive episodes [23]. In the context of suicidal behavior, systemic inflammation may further amplify negative cognitive bias, emotional dysregulation, and impulsivity, thereby elevating SA risk. Second, from an oxidative stress perspective, excess atherogenic lipoproteins promote lipid peroxidation, generating reactive oxygen species (ROS) that impair neuronal membrane integrity and prefrontal cortical function, a region critically implicated in impulse control and top‐down emotion regulation [6]. Third, with respect to neurotransmitter dysregulation, cholesterol metabolism is tightly coupled to the synthesis and membrane receptor function of serotonin (5‐hydroxytryptamine, 5‐HT); disruption of lipid homeostasis may reduce neuronal membrane fluidity and downregulate 5‐HT transporter activity, thereby attenuating the serotonergic constraint on impulsive and self‐injurious behavior [1]. Fourth, from a cerebrovascular standpoint, NHHR has been independently associated with stroke risk [24], cognitive impairment in mild stroke patients [25], and early onset poststroke depression [26], suggesting that the chronic atherogenic lipid burden accelerates cerebrovascular pathology and reduces cerebral perfusion, particularly within the prefrontal–limbic circuitry, thereby compounding suicidal vulnerability in depressed patients.

While the multilevel biological mechanisms outlined above collectively account for how elevated NHHR contributes to SA, a particularly novel and clinically significant finding of the present study is the nonlinear nature of this relationship. Curve fitting and threshold analysis revealed a significant threshold effect at NHHR = 5.76. When NHHR fell below this inflection point, each unit increment in NHHR conferred 27% greater odds of SA (*p* < 0.001), whereas at or above this threshold, the positive association became statistically nonsignificant (*p* = 0.092). The piecewise logistic regression model demonstrated a significantly superior fit relative to the conventional single‐segment model (log‐likelihood ratio test, *p* = 0.026). This threshold pattern is consistent with a growing body of evidence demonstrating that NHHR exerts context‐dependent nonlinear effects across multiple disease domains: a J‐shaped relationship with heart failure risk has been reported at an inflection point of NHHR = 2.58 [27], a J‐shaped association with peripheral artery disease at a turning point of NHHR = 2.75 [25], a nonlinear association with diabetes and prediabetes risk at a threshold of 7.09 [28], and U‐shaped or L‐shaped correlations with cardiovascular mortality across diverse populations [29]. Within the psychiatric domain, a similar nonlinear pattern of a rising‐then‐plateauing relationship between NHHR and suicidal ideation has been observed in nonsmokers, with an inflection point at 7.80 [12], and a nonlinear inverse association between ln(NHHR) and late‐life depression risk has been identified with a threshold of 0.993 [15]. Collectively, these parallel observations strongly support the premise that the biological effects of composite lipid dysregulation are inherently nonlinear and population context‐dependent.

Several mechanistic explanations may account for the attenuation of SA risk beyond the identified threshold. From a competing‐risk perspective, patients with markedly elevated NHHR are more likely to present with severe metabolic dysfunction, significant medical comorbidities, and substantial cognitive impairment [30], which may reduce volitional capacity and psychomotor drive requisite for executing suicidal behavior, thereby paradoxically attenuating the observed SA risk. This interpretation is consistent with neuroimaging evidence demonstrating that MDD with suicidality is linked to morphological and connectivity‐related abnormalities within the frontolimbic circuitry [30], which, when severely compromised, may impair the planning and execution capacity necessary for SA. Furthermore, at extremely elevated NHHR levels, qualitatively distinct neurobiological cascades associated with severe metabolic encephalopathy, including dysregulation of brain energy metabolism and neuroimmune activation beyond a critical threshold [31], may diverge from the serotonergic inflammatory pathways primarily implicated at moderate NHHR elevations, potentially redirecting neurobehavioral manifestations toward neurovegetative rather than impulsive phenotypes. Finally, patients presenting with markedly elevated NHHR in clinical settings may be more readily identified and referred for intensive psychiatric management, introducing residual confounding that cannot be excluded. These competing hypotheses require validation through prospective longitudinal studies incorporating mechanistic biomarker profiling.

The present study has several limitations. First, and most critically, the cross‐sectional design precludes any inference of the temporal sequence or causality between the NHHR and SA. Second, although the inclusion of FEU patients with MDD affords a biologically uncontaminated sample, recruitment from a single center in China limits the broader applicability of these results to other ethnic, cultural, and clinical settings. Third, despite comprehensive multivariable adjustment, potential confounding attributable to uncontrolled variables, including habitual dietary intake, physical exercise levels, genetic predispositions, circulating inflammatory markers such as C‐reactive protein, and concurrent psychosocial stressors, cannot be fully excluded. Fourth, as an observational study, the present work cannot directly elucidate the neurobiological mechanisms linking NHHR to SA; dedicated mechanistic investigations integrating inflammatory and oxidative stress and neuroimaging endpoints are therefore warranted to substantiate the proposed pathways. Furthermore, whether the observed NHHR distribution and the identified inflection point of 5.76 are consistent with nationally representative Chinese data remains to be verified; multisite replication studies across diverse geographic and clinical settings are warranted to confirm the generalizability of this threshold.

Notwithstanding these limitations, the present study provides the first evidence that NHHR is independently and positively associated with SA in FEU patients with MDD, with a clinically meaningful threshold effect at the inflection point of NHHR = 5.76. Below this threshold, every one‐unit increment in NHHR conferred 27% elevated odds for SA, positioning NHHR as a readily accessible, routinely derived lipid‐based biomarker for early SA risk stratification in this vulnerable population. Given its derivation from standard fasting lipid panels, the NHHR can be seamlessly integrated into existing clinical assessment frameworks at negligible additional costs. Further prospective longitudinal investigations are needed to establish causal directionality, confirm the identified threshold across diverse populations, and elucidate the neurobiological mechanisms through which lipid dysregulation translates into suicidal behavior in MDD.

## Author Contributions

Xiaofang Shang, Junjun Liu, and Xiangyang Zhang were responsible for study design, statistical analysis, and manuscript preparation. Diwen Shen, Xiao‐E Lang, and Xiaofang Shang were responsible for patient recruitment and data curation. Ya‐nan Sun and Junjun Liu were involved in evolving ideas and editing the manuscript.

## Funding

The authors have nothing to report.

## Disclosure

All authors have contributed to the manuscript and approved the submitted version.

## Ethics Statement

Studies involving human participants were reviewed and approved by the First Affiliated Hospital of the Shanxi Medical University (Number 2016‐Y27). Patients and participants provided written informed consent to participate in the study.

## Conflicts of Interest

The authors declare no conflicts of interest.

## Data Availability

The data that support the findings of this study are available from the corresponding author upon reasonable request.
